# Rehabilitation status of children with cerebral palsy and anxiety of their caregivers during the COVID-19 pandemic

**DOI:** 10.14744/nci.2021.32068

**Published:** 2021-12-31

**Authors:** Pinar Akpinar, Ilknur Aktas, Feyza Unlu Ozkan, Arzu Atici, Meryem Yilmaz Kaysin, Kubra Cambekli

**Affiliations:** Department of Physical Medicine and Rehabilitation, Fatih Sultan Mehmet Training and Research Hospital, Istanbul, Turkey

**Keywords:** Anxiety, cerebral palsy, COVID-19, rehabilitation

## Abstract

**OBJECTIVE:**

The purpose of the study is to explore the rehabilitation status of children with cerebral palsy (CP) and anxiety level of their caregivers during the Coronavirus disease-2019 (COVID-19) pandemic.

**METHODS:**

Caregivers of children with CP who were being followed up in our outpatient CP clinic were contacted between May 28 and June 26, 2020. Two hundred and six caregivers who voluntarily agreed to participate were administered the State-Trait Anxiety Inventory and were questioned about the rehabilitation status of their children. Demographic data, other health problems, Gross Motor Function Classification System, and Manual Ability Classification System levels of children were recorded from their files.

**RESULTS:**

All children were at home with their families during the pandemic. Their mean age was 9.58±3.84 years. One hundred and ninety-nine children were going to the rehabilitation center before the pandemic, only three children went to the rehabilitation center twice a week during the pandemic period. The anxiety level of all the caregivers was found to be high. Trait anxiety of the caregivers who did not perform home exercise to their children were found to be statistically significantly higher than those who performed exercise (p<0.05).

**CONCLUSION:**

Rehabilitation strategies should focus on reducing anxiety level in caregivers of children with CP and effective homecare therapy techniques should be acquired by the caregivers.

**O**n March 11, 2020, the World Health Organization declared Coronavirus disease-2019 (COVID-19) a global pandemic which was first reported in December 2019, in Wuhan, China [1]. Children comprise a small fraction of COVID-19 cases and show milder symptoms than adults. They are usually asymptomatic or present with fever, cough, and fatigue. Headache and gastrointestinal symptoms can be seen [2]. Dong et al. [3] reported that children who developed severe or critical COVID-19 were 10.6% of all infants, 7.3% of 1–5-year-olds, 4.3% of 6–15-year-olds, and 2.8% of 16–17-year-olds in their large (n=2135) study.

Children who have underlying medical conditions such as congenital or chronic heart, lung, and airway disease, malnutrition, immunodeficiency, hereditary metabolic conditions, and cancer are likely to become severe cases [4]. It is unknown whether certain groups for example children with comorbidities might be at a higher risk of more severe illness. Yayla et al. [5] reported that 4 out of 220 children diagnosed with COVID-19 had neurological diseases between March 11 and June 23, 2020 (intermittent lockdown period) in Turkey. Moreover, there is no data about the children with disabilities.

Cerebral palsy (CP) is the most common physical disability in childhood which affect the developing nervous system causing permanent motor, postural, and functional impairment. Children with CP require more care and supervision than the normally developing children. Caregivers of children with CP should cope with the difficulties of the disability of their children. They had a higher level of psychological and physical symptoms than the caregivers of the healthy children. Long-term responsibility for a disabled child and challenges associated with handling the child often leads to anxiety, distress, anger, sadness, and hopelessness experienced by the caregiver. It has been shown that the quality of life and psychological status in mothers of children with CP are worse as compared to mothers of healthy children. Since active participation of the mother in the rehabilitation of a child with CP can ensure reaching the goals of therapy, protection of mental and physical health of mother is rather important [6, 7].

The COVID-19 outbreak has also brought emotional distress and psychological and social difficulties. The risk of transmission of the virus to the families and their children may increase the anxiety level of families [8]. Recently published studies stated that the current pandemic has led to stressful situations for people and communities [9]. A general worsening of sleep quality and distortion of time experience in both mothers and children, as well as increasing emotional symptoms and self-regulation difficulties in children, were observed [10]. The ECHO French survey investigated the healthcare issues of disabled children, continuity of rehabilitation and medical care, and parental concerns during the COVID-19 lockdown. Rehabilitation services were massively interrupted, and this was the main parental concern. Parents were faced with the burden of managing the child’s daily life as well as providing rehabilitation [11].

Due to the lockdown, disabled children could not attend to their physiotherapy sessions. We expect that determining how children with CP, a population that requires special support, spent this period and the level of difficulties and anxiety experienced by caregivers, will contribute to the treatment protocols for this population. Hence, we planned this study.

**Highlight key points**

• Rehabilitation programs of children with CP were interrupted during the COVID-19 pandemic.
• The anxiety levels of all caregivers were high during the COVID-19 pandemic.
• Effective home care therapy techniques should be learned by the caregivers of children with CP.

## Materials and Methods

Children who were followed in private outpatient CP clinic of physical medicine and rehabilitation department in Fatih Sultan Mehmet Training and Research Hospital were contacted by phone and invited to the study from 28^th^ May to 26^th^ June 2020. All procedures were conducted in accordance with the Helsinki Declaration of 1975 and were approved by the Fatih Sultan Mehmet Training and Research Hospital Research Ethics Committee with a protocol number of FSMEAH-KAEK 2020/56. Clinicaltrials.gov registry number is NCT04916873.

**Figure 1.**
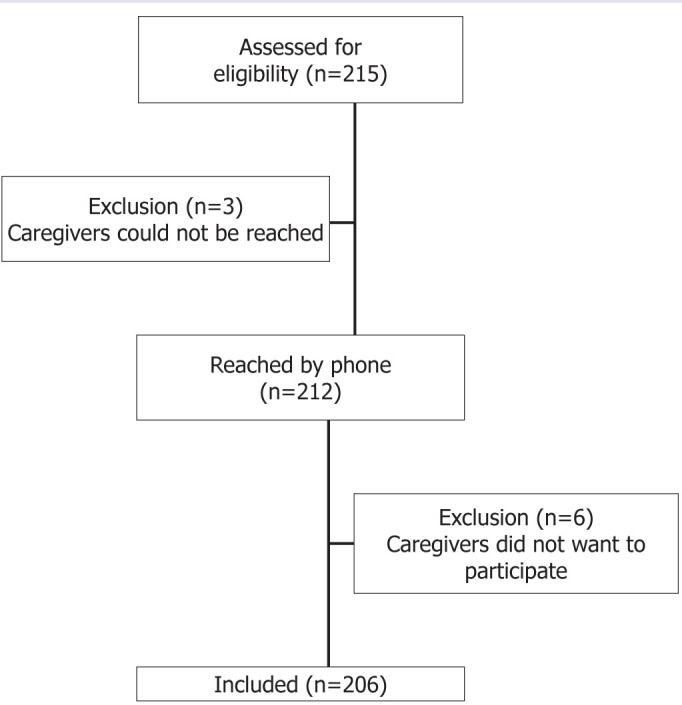
Study flow diagram.

Of the 215 caregiver, 212 were reached by phone and 206 accepted to participate in the study. Three caregivers could not be reached and six caregivers did not want to participate in the study. Study flow diagram is presented in Figure 1. Demographic data and CP types of children, other health problems, levels of the Gross Motor Function Classification System (GMFCS), and the Manual Ability Classification System (MACS) were recorded from the existing files of the most recent follow-up visit. Cargivers were questioned on where and with whom the children went through the pandemic period, their rehabilitation processes, whether they received additional medical treatment, whether there was any deterioration in their general condition, and whether family members were diagnosed with COVID-19. The State-Trait Anxiety Inventory form (STAI) was administered to the caregiver by structured telephone call.

STAI, developed by Spielberger et al. [12], consists of two likert-type subscales that separately measure state and trait anxiety levels with 20 questions. The State Anxiety Scale (STAI-1) determines how a person feels at a particular moment and condition, while the Trait Anxiety Scale (STAI-2) determines how the person feels regardless of the situation and condition he or she is in. In STAI-1, according to the severity of the emotions, thoughts or behaviors expressed by the items, “nothing,” “a little,” “a lot,” and completely “, in STAI-2, the frequency of the emotions, thoughts or behaviors expressed by the items, “almost never,” “sometimes,” a lot of time “and” almost always “ as scored at 4 degrees. The total score of both scales varies between 20 and 80. Those who score below 36 are classified as “no anxiety,” those between 37 and 42 points as “mild anxiety” and those with 42 points and above as “high anxiety.” The scale was translated into Turkish and adaptation, validity, and reliability studies were performed [13].

GMFCS is a widely used standard measurement tool to classify the gross motor functions of children with CP [14]. It consists of five levels; level I indicates independent mobility, level V indicates full dependency. The Turkish version was developed by Gunel et al. [15], and its reliability was demonstrated by El et al. [16]

MACS is a 5-level scale that classifies the ability of children with CP to use their hands in daily activities, ranging from level I (highest abilities) to level V (the most limited abilities), according to their age. It was developed by Eliasson et al. [17, 18] in 2006 and the mini-MACS was developed in 2017 for children under 4 years of age. Cross-cultural adaptation was done to the Turkish population by Akpinar et al. [19]

### Statistical Analysis

For the statistical analysis, IBM SPSS Statistics 22 (SPSS IBM, Turkey) program was used. G*Power was used for the calculations of the estimated sample size which was based on a previous study by Erkek and Aktas assuming that α is 0.05 and power is 0.80 [20]. Minimum sample size of 142 subjects was determined. While evaluating the study data, the suitability of the parameters to the normal distribution was evaluated with the visual (histogram, etc), Kolmogorov-Smirnov, and Shapiro Wilks test. In addition to descriptive statistical methods (mean, standard deviation, frequency), the Oneway Anova test was used for the comparison of parameters showing normal distribution between groups in the comparison of quantitative data, and the Tukey HDS test was used to identify the group that caused the difference.

Student t-test was used for comparisons of normally distributed parameters between two groups. Fisher’s Exact test, Chi-square test, Fisher Freeman Halton test, and Continuity (Yates) Correction were used in the comparison of qualitative data. Pearson’s correlation analysis was used to examine the relationships between parameters suitable for normal distribution. Significance was evaluated at the p<0.05 level.

## Results

A total of 206 children, 77 (37.4%) girls and 129 (62.6%) boys, and their caregivers participated in this study. The ages of the children were between 2 and 18 and the mean was 9.58±3.84 years, while the age of the caregivers was between 22 and 49 and the average was 32.67±5.82 years. CP types of children, GMFCS and MACS levels, other health problems, rehabilitation frequency of children before the pandemic, and education and monthly income levels of caregivers are shown in Table 1. STAI levels of the caregivers are presented in Table 2. Fifty-one point five percent of the caregivers had a high level of state anxiety, while 59.7% had a high level of trait anxiety during the pandemic period.

**Table 1.** CP types of children, GMFCS and MACS levels, associated problems, education status of caregivers, and monthly income levels

n=206	%
CP type	
Spastic Unilateral (Hemiplegic)	26.7
Spastic Bilateral	
Diplegic	35
Quadriplegic	21.8
Triplegic	13.1
Dyskinetic	1
Ataxic	0.5
Mixed	1.9
GMFCS level	
1	10.7
2	30.6
3	27.2
4	20.9
5	10.7
MACS level	
1	16.5
2	36.9
3	18
4	14.1
5	14.6	Associated problems
Present	84.5
None	15.5
Associated problems	
Speech impairment	55.3
Mental retardation	46.1
Epilepsy	31.1
Respiratory problems	5.8
Visual impairment	25.7
Strabismus	42.2
Hearing impairment	4.4
Behavioral problems	12.1
Difficulty in swallowing	18
Growth retardation	23.3
Dental problems	21.8
Drooling	18.4
Rehabilitation status	
None	3.4
1–2 day/week	50.5
3–4 day/week	39.8
5–6 day/week	6.3
None	4.4
Primary school	58.7
High school	27.2
University	9.7
Monthly income	
Minimum wage	56.8
Minimum wage×2	36.4
Minimum wage×3	5.8
Minimum wage×4	1

CP: Cerebral palsy; GMFCS: Gross motor function classification system; MACS: Manual ability classification system; n: Number.

**Table 2.** STAI parameters

	Min-Max	Mean±SD
STAI-1 (State)	21–76	43.4±10.5
STAI-2 (Trait)	25–73	43.8±7.4
STAI total	50–149	87.2±16.2
	n	%
State anxiety scale (STAI-1)		
No anxiety	61	29.6
Mild anxiety	39	18.9
High anxiety	106	51.5
Trait anxiety scale (STAI-2)		
No anxiety	33	16
Mild anxiety	50	24.3
High anxiety	123	59.7

STAI: State-trait anxiety inventory; SD: Standard deviation.

All of the children were at home with their families during the pandemic period. Primary caregiver is the mother in 203 children, only in three children, father is the primary caregiver with the help of the grandmother. Forty-two (20.4%) children had to postpone their routine doctor follow-up, 26 (12.6%) children had to postpone their botulinum toxin injection appointments but all children were able to take their antiepileptic or antispastic medications. One hundred twelve (54.4%) caregivers stated that there was a deterioration in the general condition and spasm (spasticity) of their children. Thirty-six (17.4%) of the caregivers used a supplementary vitamin/herbal product to protect their children from COVID-19. While 17 caregivers preferred herbal supplements and food, nine used Vitamin D, and six used multivitamin. The rate of using supplements for their children to be protected from COVID-19 infection was found to be statistically significantly lower in caregivers with primary education than those with high school and university degrees (p<0.05).

There were 39 families, who were diagnosed with COVID-19 in their immediate vicinity such as neighbors, colleagues, relatives. In one family, the father was diagnosed with COVID-19. There wasn’t any statistically significant difference in STAI parameters between those who were diagnosed with COVID-19 in their immediate vicinity and those who were not diagnosed with COVID-19 in their immediate vicinity (p>0.05).

While 199 children were going to the rehabilitation center before the pandemic, only three children went to the rehabilitation center once a week during the pandemic period due to the intermittent lockdown. Twenty-three (11.2%) children did not exercise at home during the pandemic, 86 (41.7%) exercised regularly (at least 3 times a week for 30 min), 97 (47.1%) exercised irregularly. While 35 (19.1%) caregivers thought that they performed the exercise effectively, 148 (80.9%) caregivers thought that they could not perform it effectively.

The correlation of the STAI parameters with the ages of children and caregivers is shown in Table 3. There was a positive statistically significant correlation only between the children’s ages and the STAI-1 scores (p<0.05).

**Table 3.** Correlation of the STAI parameters with the age of the children and their caregivers

	Child age	Caregiver age
STAI-1		
r	0.15	0.08
p	0.02*	0.23
STAI-2		
r	0.06	–0.04
p	0.32	0.55
STAI total		
r	0.13	0.03
p	0.06	0.60

*: P<0.05. STAI: State-trait anxiety inventory.

The evaluation of STAI parameters according to the child’s gender, GMFCS and MACS levels, and the educational status and monthly income level of the caregiver are shown in Table 4. STAI-1 and total STAI scores of caregivers who have a child with GMFCS level 1 were found to be statistically significantly lower than the caregivers who have a child with GMFCS level 4 and 5 (p<0.05). The STAI-1 scores of the caregivers who have a child with MACS level 1 were found to be statistically significantly lower than the caregivers who have a child with MACS level 2, 4, and 5, and the STAI total scores were found to be statistically significantly lower than the caregivers who have a child with MACS level 4 and 5 (p<0.05).

**Table 4.** Evaluation of STAI parameters according to the child’s gender, GMFCS and MACS levels, education level of the caregiver, and monthly ıncome levels

n=206	STAI-1 Mean±SD	STAI-2 Mean±SD	STAI total Mean±SD
Gender			
Girl	44.5±10.9	43.5±7.8	88.0±16.7
Boy	42.8±10.3	44±7.2	86.8±15.9
p	**0.275**	**0.645**	**0.616**
GMFCS level			
1	36.6±8.0	41.2±6.3	77.9±11.2
2	42.6±9.7	43.1±6.5	85.7±13.8
3	43.5±11.3	43.4±6.7	87.0±16.3
4	46.4±11.3	45.8±9.3	92.2±19.5
5	46.5±8.9	45.2±7.3	91.7±15.3
p	0.005*	**0.119**	0.008*
MACS level			
1	37.6±7.4	41.4±6.0	79.1±11.6
2	43.5±11.0	42.9±6.6	86.4±15.2
3	44.2±10.3	44.7±7.8	88.9±16.8
4	46.1±10.0	45.6±7.6	91.7±16.6
5	46.2±11.3	45.8±9.0	92.1±18.8
p	0.006*	**0.054**	0.006*
Education level of caregiver			
None	37±4.03	42.8±6.1	79.8±6.7
Primary school	44.8±10.8	44.9±7.6	89.7±16.7
High school	42.2±10.2	42.0±6.2	84.2±14.7
University	41.9±10.9	42.6±8.7	84.6±17.5
p	**0.088**	**0.086**	**0.065**
Monthly income			
Minimum wage	44.5±10.7	44.9±7.5	89.4±16.5
Minimum wage×2	41.4±9.7	42.0±6.8	83.5±14.7
Minimum wage×3×4	42.4±10.7	42.1±7.9	84.5±15.3
p	**0.143**	0.021*	0.038*

*: P<0.05. GMFCS: Gross motor function classification system; MACS: Manual ability classification system; STAI: State-trait anxiety inventory; SD: Standard deviation; n: Number.

The STAI-2 and total STAI scores of those whose monthly income was within the ranges of the minimum wage were found to be statistically significantly higher than those with the twice the minimum wage (p<0.05).

When other associated problems of children were examined, the total STAI and STAI-1 scores of the caregivers who have a child with speech impairment and mental retardation were found to be statistically significantly higher than those who have a child without these problems (p<0.05). All STAI scores of caregivers who have a child with behavioral problems were statistically significantly higher than those who have a child without behavioral problems. STAI-2 and total STAI scores of caregivers who have a child with respiratory problems were found to be statistically significantly higher than those who have a child without respiratory problems (p<0.05).

All STAI scores of caregivers who thought that there was a worsening in their children’s general condition and contractions were found to be statistically significantly higher than those who did not think that there was a worsening (p<0.05).

The STAI-2 scores of the caregivers who performed home exercise to their children with CP during the pandemic period were found to be statistically significantly lower than those who did not perform home exercise. All the STAI scores of the caregivers who thought that they performed the home exercise effectively were found to be statistically significantly lower than the caregivers who thought that they could not perform the exercise effectively. The average age of caregivers who performed home exercise to their children during the pandemic period was found to be statistically significantly lower than those who could not perform exercise (p<0.05).

## Discussion

Entire world is greatly struggling with the COVID-19 pandemic, which affects the society physically, socially, economically, and psychologically. In addition to the medical problems that this virus directly causes, the contagiousness of the disease, the possibility of contamination of the virus to the loved ones, uncertainties in treatment, isolation due to quarantine, measures taken, economic problems, and uncertainties about the future cause mental problems such as anxiety, fear, panic, and depression in people [9, 21]. Due to the pandemic, the services of individuals who require regular healthcare and the rehabilitation of disabled patients have also been disrupted [22]. In this study, we investigated how children with CP, a population that requires special support, spent this period and anxiety levels of their caregivers, it was found that the regular rehabilitation of the children were interrupted and anxiety levels of all caregivers were found to be high.

All children with CP were at home with their families through the pandemic, this may have reduced the COVID-19 exposure rates seen in this study. Only one father was diagnosed with COVID-19, and there were 39 families diagnosed with COVID-19 in their immediate vicinity. Cankurtaran et al. [23] have found 5.3% of the children and 13.8% of the caregivers had a history of COVID-19 infection in their study where they have evaluated the effects of the COVID-19 pandemic on children with CP. They stated that most of the children had to take a break in their physical therapy sessions similar to our study only three children went to the rehabilitation center during the pandemic period due to the intermittent lockdowns.

Caregivers had a high level of anxiety. In this study, 51.5% of them had a high level of state anxiety and 59.7% of them had a high level of trait anxiety. Anxiety, which each individual may experience in different periods and intensities in his or her life, is divided into “State Anxiety” and “Trait Anxiety.” State anxiety is a temporary form of anxiety that occurs at a particular moment and event, usually with logical reasons. Regardless of any situation or event, general and continuous anxiety is called trait anxiety [13]. It is known that the trait anxiety of mothers who have disabled children was also high before the pandemic [24]. During the pandemic period, it was also found that women and especially women with children under 16 years of age had a high level of anxiety in the general population [25].

In their letter to the editor, which explains their first experience in Italy, Elisa Fazzi, and Jessica Galli stated that the COVID-19 epidemic causes high levels of stress, anxiety, and depression in children with disabilities and their families, and call centers offered psychological and psychiatric support to both children and their families [26]. Farajzadeh et al. [27] investigated the psychological health of Iranian caregivers of children with CP and associated risks during the lockdown period. Burden of care, low income, and low educational level were found to be the main predictors of anxiety. In this study, we found a relationship between the anxiety of the caregivers and income levels but we did not find any relationship between the level of education. A comprehensive plan including psychological consultation, remote education, and adequate distance support for these caregivers was recommended.

Moreover, studies have found that caregivers who do not use telerehabilitation for care of their children with special needs have high anxiety levels during the COVID-19 pandemic. In addition, a strong association was found between the caregiver’s consensus against teleconsultation as an alternative tool for rehabilitation and psychological symptoms [8]. In this study, the trait anxiety of caregivers who performed home exercise to their children with CP during this period was found to be statistically significantly lower than those who did not perform home exercise. Both state and trait anxiety of caregivers who thought that they performed the home exercise effectively were found to be statistically significantly lower than those who thought that they could not perform the exercise effectively. Therefore, home exercise programmes should be supported.

There are many studies suggesting that parental psychic suffering impairs health care and decreases the rehabilitation and development performance of the children with CP before the pandemic [6]. Factors such as the functional level of the child and socioeconomic status can affect the frequency of mental disorders in parents [6, 28]. In this study, the state anxiety and state-trait anxiety of the caregivers who have a child with CP with worse gross motor function and hand function were found to be higher than those who have a child with better motor functions. Children with poor motor function have more associated problems, are more dependent, and need more support. This may create more anxiety for the caregiver, restrict caregiver’s own needs and adversely affect their psychology.

Caregivers of children with CP who have speech impairment, behavioral problems, and mental retardation were found to have higher levels of state and trait anxiety, than the caregivers of children without these problems. We think that the inability to communicate with the child might have increased the anxiety level of the caregiver in this period. However, we can not draw such a conclusion with the current data, since, the state-trait anxiety of the caregivers before and after the pandemic were not compared, which is a limitation of this study.

Another limitation of this study was that we coud not evaluate the physical and psychological status of these children because of the lack of a valid and reliable assessment scale to evaluate the joint movements of children, muscle tonus, and stress which can be administered through phone call. Furthermore, a study comparing anxiety in caregivers of children with CP and caregivers of normally developing children would be useful.

Recently, family-focused therapies are considered as one of the best service models in carrying out the rehabilitation of the child by putting the family in the center. Less depression and anxiety have been reported in these families [29]. In addition, it is stated that continuing the rehabilitation at home can be more effective in increasing the functional skills of the child by practicing in the natural environment and by increasing the amount of training [30]. Due to the special conditions and precautions taken during the pandemic period, caregivers had to apply rehabilitation techniques at home. In this study, 88.8% of the caregivers performed home exercise to their children, 41.7% of them stated that they performed the exercise regularly and 19.1% of them stated that they performed the exercise effectively. Therefore, especially in this period, telerehabilitation can replace face-to-face applied rehabilitation and reduce the stress and burden on caregivers. With the advances in technology, telerehabilitation is applied as an alternative method in health services [8].

As a result, staying at home protected children with CP from COVID-19 exposure, but their rehabilitation programs were interrupted. Hence, effective homecare therapy techniques must be learned by the caregivers and the situation of the child and caregiver must be regularly re-evaluated by the healthcare professionals during the pandemics. Anxiety and tension in caregivers of children with CP should also be reduced. With multidisciplinary support, proper communication, and interaction between healthcare professionals and families, children with CP can receive better care during current and future pandemics.
